# Low health literacy levels in patients with chronic retinal disease

**DOI:** 10.1186/s12886-019-1191-1

**Published:** 2019-08-08

**Authors:** Sofie Jandorf, Marie Krogh Nielsen, Kristine Sørensen, Torben Lykke Sørensen

**Affiliations:** 1 Clinical Eye Research Division, Department of Ophthalmology, Zealand University Hospital, Vestermarksvej 23, DK-4000 Roskilde, Denmark; 2 Global Health Literacy Academy, Risskov, Denmark; 3 Faculty of Health and Medical Science, University of Copenhagen, Copenhagen, Denmark

**Keywords:** Age-related macular degeneration, Diabetic macular edema, Health literacy, Retinal vein occlusion

## Abstract

**Background:**

A patient’s health literacy is fundamental for navigating the health system and managing disease. This study aimed to compare the health literacy levels of patients with chronic retinal disease in Denmark.

**Methods:**

This cross-sectional questionnaire study used the validated HLS-EU-Q16 questionnaire to determine the health literacy of 225 patients with age-related macular degeneration (AMD), diabetic macular edema (DME) or retinal vein occlusion (RVO), receiving intravitreal treatment at the retinal clinic, Zealand University Hospital, Denmark. Patients were consecutively included as participants for the study. All patients had the option of having the survey read aloud to them.

**Results:**

Health literacy levels between the patient groups did not differ significantly, however, the proportion of patients with poor health literacy was high—65% of AMD patients, 73% of DME patients, and 63% of patients with RVO.

**Conclusions:**

Low health literacy of patients with retinal disease signify a need for more health literacy research in the field of retinal diseases, to secure that patients have the timely and appropriate knowledge and competencies to manage their condition.

## Background

Health literacy entails the knowledge, motivation and competency to access, understand, appraise and apply information concerning healthcare, disease prevention and health promotion. [1] Hence, it relates to patients’ ability to understand and monitor their disease as well as navigate the health system. Health literacy has important implications for the health care system—patient, physician and organization [2] and is essential to a person’s overall health and disease prognosis as it influences their ability to self-medicate and make health-related decisions. [2–4] Poor health literacy may contribute to poor outcomes, as low health literacy has previously been linked to worse disease prognosis, especially in patients with chronic conditions. [5, 6] In type 2 diabetes, poor health literacy level is associated with increased risk of complications, such as diabetic retinopathy. [6] Further, in patients with glaucoma, poor health literacy skills are associated with lower adherence to treatment with glaucoma medications [3] and associated with worsening of the visual field. [2] Patients with low vision tend to spend more time and have greater difficulty reading health-related materials, which can induce lower health literacy scores. [4] Muir et al. suggested that by providing patient education appropriate for patients with low health literacy, eye care providers have the opportunity to improve clinical outcomes and reduce healthcare disparities. [7]

Research on health literacy has been expanding in recent years. However, this study is of importance, as health literacy research is scarce in the area of ocular diseases.

The purpose of this study is to understand and compare the health literacy levels of patients with chronic retinal diseases; age-related macular degeneration (AMD), diabetic macular edema (DME), and retinal vein occlusion (RVO).

## Methods

### Participants and study design

This cross-sectional study comprises patients with clinical diagnosis of AMD, DME or RVO in at least one eye, receiving intravitreal treatment with anti-vascular endothelial growth factor (VEGF) in the period between August and December 2018. Adult Danish-speaking patients of both genders without any known severe mental disorders or dementia were eligible for participation. We explained the nature of the study to all patients and verbal informed consent was obtained prior to their participation.

Patients were selected on a first-come basis, as the interviewer included the first patient arriving at the department from when the interviewer was available. The study complies with the tenets stated in the Declaration of Helsinki. Since the study includes data from a questionnaire, it is by Danish law (Komitéloven § 14, stk. 2) exempted from registration at the Regional Research Ethics Committee.

### Patient characteristics

Patients were interviewed regarding education level, self-perceived health, comorbidities, and postal code. Education levels were based on the highest achieved level of education, defined as described by the Danish Ministry of Education. The levels of education were: primary school, vocational, short-cycle higher education (approximately two years at Academy profession programmes), medium-cycle higher education (approximately 3.5 years at Professional Bachelor’s programme), and long-cycle higher education (approximately 5 years at University programme). [8]

Postal codes were classified by municipalities: peripheral, rural, intermediate, or urban as suggested by the Danish Ministry of Food, Agriculture and Fisheries. [9]

Best corrected visual acuity (VA) was measured based on the Early Treatment Diabetic Retinopathy Study (EDTRS)-protocol by trained nurses. [10] The patient is scored by how many letters are correctly identified, after reading from the top of the chart. Patients read down the chart until a minimum of three letters cannot be read. Further, we converted the ETDRS value into the logarithm of minimum angle of resolution (LogMAR) from each patient. [11]

Comorbidities were converted based on the Charlson’s Comorbidity Index (CCI). CCI applies comorbidities into four categories; none (0), low (1–2), medium (3–4) or high (> 4). [12, 13] Diabetic maculopathy was not registered as a diabetic complication in this index, to better assess other disorders besides the known retinal disease.

### Health literacy assessment

The European Health Literacy Survey Questionnaire (HLS-EU-Q47) was developed to measure self-perceived health literacy across Europe [14, 15] and was translated into Danish and validated by Maindal et al. [16] The short form called HLS-EU-Q16 consists of 16 items focusing on four health literacy dimensions reflecting perceived ease or difficulty in an individual’s ability to obtain, understand, process, and apply health information.

A Danish-speaking physician or nurse administered the survey and patients got the option of having the survey read aloud to them accommodating patients at all vision and literacy levels.

The responses were dichotomized with “very easy” and “easy” given a score of 1 and “difficult” and “very difficult” given a score of 0.

The scores of the patients were then divided into three qualitative categories of health literacy: ‘inadequate’ (1–8), ‘problematic’ (9–12), ‘sufficient’ (13–16). [17]

### Statistical analyses

Statistical analysis were performed using the SPSS version 24 (IBM Corporation, Armonk, NY, USA). Normally distributed data was presented in mean and standard deviation (SD) and compared using parametrical tests. Not normally distributed data were presented using median and interquartile range (IQR) and compared using non-parametrical tests. We used Spearman rank correlation to assess possible correlations. Categorical data were tested using χ^2^ test, unless sample size was small (< 5 cases) in which case Fisher’s exact test was used.

## Results

A total of 225 patients completed the questionnaire. Of these, 22 patients were excluded as they completed less than 80% of the questionnaire. Therefore, a total of 203 patients composed our study sample, distributed as following: 145 patients with AMD, 26 with DME and 32 with RVO.

### Patient characteristics

Patient demographics, clinical data, and self-perceived health are shown in Table 1. The patient groups differed in terms of age, since patients with DME were younger (*P* < 0.001). Also, participants of this study represent more females with AMD and fewer females with DME (*P* = 0.001). Patients with AMD was treated with anti-VEGF for a longer period of time than patients with DME and RVO. Patients with DME had a higher frequency of comorbidities (*P* < 0.001), a higher frequency of poor self-reported health (*P* = 0.013), and an overall higher education level (*P* = 0.007) than the other groups.

**Table 1:** Characteristics of participants in sociodemographic factors, visual acuity, duration of treatment, and comorbidities

	AMD, *n* = 145	DME, *n* = 26	RVO, *n* = 32	*P*-value
Age, mean (SD)	77.4 (7.4)	61.8 (13.0)	74.5 (9.9)	< 0.001^a^
Gender, *n* (%)				
Female, *n* (%)	94 (64.8)	7 (26.9)	17 (53.1)	0.001^b^
Male, *n* (%)	51 (35.2)	18 (73.1)	15 (46.9)	
Visual acuity in best eye, ETDRS, median (IQR)	80.0 (72–85)	75.5 (65–85)	82.5 (74–85)	0.246^c^
Visual acuity in best eye, LogMAR, median (IQR)	0.1 (0.0–0.3)	0.2 (0.0–0.4)	0.0 (0.0–0.2)	
Treatment duration in years, median (range)	2 (0–11)	2 (0–6)	1 (0–5)	0.014^c^
Classes of municipalities				0.749^d^
Urban	45 (31.0)	6 (23.1)	11 (34.4)	
Intermediate	56 (38.6)	9 (34.6)	9 (28.1)	
Rural	38 (26.2)	9 (34.6)	10 (31.3)	
Peripheral	6 (4.1)	2 (7.7)	2 (6.3)	
Education level, *n* (%)				0.007^d^
Long-cycle higher education	9 (6.3)	5 (19.2)	0 (0)	
Medium-cycle higher education	26 (18.1)	4 (15.4)	11 (34.4)	
Short-cycle higher education	37 (25.7)	1 (3.8)	7 (21.9)	
Vocational	36 (25.0)	12 (46.2)	8 (25.0)	
Primary school	36 (25.0)	4 (15.4)	6 (18.8)	
Self perceived health, *n* (%)				0.013^d^
Good	68 (47.2)	15 (57.7)	12 (37.5)	
Average	66 (45.8)	5 (19.2)	18 (56.3)	
Poor	10 (6.9)	6 (23.1)	2 (6.3)	
Charlson’s Comorbidity Index, *n* (%)				< 0.001^d^
None (0)	85 (58.9)	0	21 (65.6)	
Low (1–2)	50 (34.5)	21 (80.8)	11 (34.4)	
Medium (3–4)	9 (6.2)	3 (11.5)	0	
High (> 4)	1 (0.7)	2 (7.7)	0	

Abbreviations: AMD: Age-related macular degeneration; DME: Diabetic macular edema; RVO: Retinal vein occlusion; n: number; SD: standard deviation; EDTRS: Early Treatment Diabetic Retinopathy Study; LogMAR: Log of the minimum angle of resolution; IQR: interquartile range; ^a^One-way ANOVA; ^b^Chi square Test; ^c^Kruskal-Wallis Test; ^d^Fishers Exact Test

### Health literacy

**Fig. 1:**
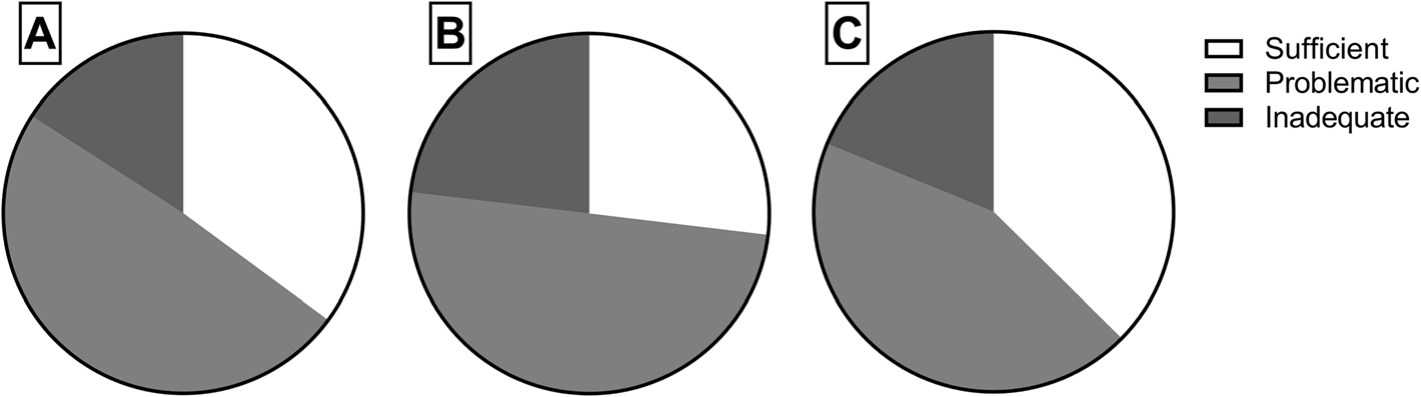
The majority of patients with age-related macular degeneration (A), diabetic macular edema (B), or retinal vein occlusion (C) had problematic or inadequate health literacy level

The proportion of patients with inadequate or problematic health literacy was high—65% of patients with AMD, 73% of DME patients, and 63% of patients with RVO had problematic or inadequate levels of health literacy (Fig. 1). Health literacy levels did not differ between patient groups (*P* = 0.819).

Self-perceived health showed a significant association with health literacy levels, as patients reporting “good” health, had an increased chance of sufficient health literacy. (Odds ratio: 3.02; 95% CI: 1.14–7.99).

There was a tendency towards higher levels of health literacy in patients with a medium cycle or long cycle of higher education level (*P* = 0.036).

We did not find any association between health literacy levels and: age (*P* = 0.204), gender (*P* = 0.284), visual acuity (*P* = 0.730), treatment duration (*P* = 0.195), municipalities (*P* = 0.587), or the number of comorbidities in CCI (*P* = 0.429).

## Discussion

In this study, we found that the majority of patients with chronic retinal disease have an inadequate or problematic level of health literacy. Notably, we found no difference in health literacy between diagnosis groups, but an overall low health literacy score.

The relationship between health literacy and ocular diseases is unique as health literacy, among other parameters, depends on the level of vision. Warren et al. found that older adults with low vision had considerably lower health literacy than older without low vision. [4] Studies on health literacy in ophthalmic diseases are highly relevant as our current healthcare system is predominantly designed to be navigated visually and includes basic requirements such as reading prescription bottles, informational documents, online materials, and appointment forms. In this study, we included patients with retinal disease, regardless of low vision or good vision. Many of our participants were only affected in one eye, and all patients were receiving relevant treatment. Therefore, we do not investigate the association between low vision and health literacy, but merely describe a population of patients suffering from retinal disease. [4, 18]

A chronic retinal disease requires self-monitoring, self-medication, and self-care. [2–4, 6] Therefore, health literacy may affect disease prognosis and interact in a feedforward loop causing an amplification of adverse effects: Poor health literacy could lead to poor compliance and lacking adherence to the treatment schedule, further leading to a poor outcome. This is the case in other chronic medical diseases, such as diabetes, where lower health literacy is associated with a higher occurrence of microvascular complications. [19] Studies describing health literacy in the course of retinal disease are needed to uncover if a similar relationship exists.

One of the most significant risk factors for chronic diseases overall is low economic status. [6] Low economic status has also been strongly correlated with low health literacy scores. [7, 20] The structure of the Danish healthcare system is required to provide universal care for all citizens, including transportation from home address, diminishing the possible selection bias of patient’s seeking the retinal clinic to receive relevant treatment. To address the risk of selection bias is of particular relevance to the field of health literacy as patients with low health literacy tend to visit the doctor more frequently and when it is not needed, resulting in a discrepancy between the need and demand for health services. [21] To overcome the challenge related to low health literacy, guidance and education have the potential to enable and empower patients to sustainably manage their health. Parker & Ratzan suggest a two-sided approach to do so: 1) strengthen patients’ personal knowledge, motivation, and competencies to make well-informed health decisions, and 2) decrease complexity of systems and society as a whole [22] In turn, building on the approach of Parker & Ratzan, Sørensen proposes to enhance people-centered care by increasing the skills of health organizations and systems to meet the complex demands of people. [23] In this respect, user-friendly and user-involving systems can be designed to strengthen health literacy. Educating health professionals about the low levels of health literacy among patients seen in the retinal clinic might improve their abilities to better meet the patients’ needs and demands when taking into account their health literacy.

We found that poor health literacy was associated with poor self-perceived health. This is in contradiction to the fact that a high CCI, was not associated with lower health literacy in this patient group. This is of particular interest, as it indicates that low levels of health literacy might have consequences, such as inadequate understanding of disease mechanisms leading to anxiety and poor self-perceived health.

Research reveals that health literacy leads to improved self-reported health status, lower healthcare costs, increased health knowledge, shorter hospitalization, and less frequent use of healthcare services. [1] Increasing nurses’ and ophthalmologists’ understanding of their patients’ health literacy levels would allow them to better target specific areas in order to ensure efficient and effective treatment for their patients and potentially prevent poor visual outcomes.

It is important to acknowledge the limitations of this study, which include the respondents’ understanding of the questions, cultural/ethnicity differences and emotional state of the respondents upon answering the questions, which could not be controlled by the investigator. Further, this study does not allow to study the influence of low vision, as we included all patients regardless of visual acuity. In planning future studies, we suggest to include information on what percentage of patients were in need of having the survey read to them, or the use of magnification aid. Also, low vision rehabilitation might influence on health literacy. There is a possible selection bias from participants being patients at a Danish research hospital, limiting the extent to which these results can be applied.

## Conclusion

In conclusion, we find that many patients with chronic retinal disease have a low level of health literacy, which signifies a need for more health literacy research in the field of ocular diseases. Further research is needed to investigate the potential consequences of poor health literacy in the individual patients’ course of disease.

## Data Availability

The datasets used during the current study are available from the corresponding author on reasonable request.

## Abbreviations

AMD: Age-related macular degeneration
CCI: Charlson comorbidity index
DME: Diabetic macular edema
EDTRS: Early Treatment Diabetic Retinopathy Study
LogMAR: Logarithm of minimum angle of resolution
RVO: Retinal vein occlusion
VA: visual acuity
VEGF: vascular endothelial growth factor
